# Allergen Risk Assessment for Specific Allergy to Small Ruminant's Milk: Development of Sensitive Immunoassays to Detect Goat's and Sheep's Milk Contaminations in Dairy Food Matrices

**DOI:** 10.3389/falgy.2021.733875

**Published:** 2021-09-30

**Authors:** Hervé Bernard, Stéphane Hazebrouck, Nicolas Gaiani, Karine Adel-Patient

**Affiliations:** Université Paris-Saclay, CEA, INRAE, Département Médicaments et Technologies pour la Santé, Service de Pharmacologie et d'Immunoanalyse, Laboratoire d'Immuno-Allergie Alimentaire, Gif-sur-Yvette, France

**Keywords:** milk, allergy, sheep, goat, IgE, β-casein, ELISA, dairy product

## Abstract

Despite a high level of sequence identity between cow's, goat's, and sheep's milk (CM, GM, and SM, respectively) proteins, some patients tolerant to CM are allergic to GM and SM. In most cases, this specificity is due to the presence of IgE antibodies that bind only to caprine and ovine caseins. The patients may then develop severe allergic reactions after ingestion of CM products contaminated with low amounts of GM or SM. We thus aimed to develop an assay able to detect traces of caprine/ovine β-caseins in different food matrices, irrespective of the presence of the bovine homolog. We produced monoclonal antibodies (mAbs) specific to caprine caseins in mice tolerized to the bovine whole casein then sensitized to the caprine whole casein. In order to develop a two-site immunometric assay, we selected mAbs that could discriminate the caprine β-casein from its bovine homolog. Characteristics and performances of two tests were determined with various dairy products. Results were analyzed in relation with the IgE-immunoreactivity of the food matrices, thanks to sera from CM, GM/SM allergic patients. Our two-site immunometric assays demonstrated a high sensitivity with a detection limit of 1.6–3.2 ng/mL of caprine and ovine β-caseins. The tests were able to detect contaminations of GM in CM at the ppm level. Heat-treatment, ripening and coagulation processes, usually applied to dairy products that exhibit a very high IgE-immunoreactivity, did not impair the test sensitivity. These quantitative assays could then be useful for the risk assessment of food products potentially contaminated with GM and SM in order to prevent adverse reactions in patients specifically allergic to these milks.

## Introduction

Milk is one of the foods causing the most frequently an adverse reaction (1, 2). Cross-allergenicity between milks from different ruminants are generally observed, thus rendering the use of goat's and sheep's milks (GSM) as substitute of cow's milk (CM) unsafe for patients allergic to CM (3–5). This cross-allergenicity results from similar protein compositions and from a high sequence homology between protein components of ruminant milks (6–8). Nevertheless, despite these structural similarities, allergic reactions to GM or SM can occur in patients tolerant to CM. Since the first report by Wuthrich, this allergy has been regularly observed, mainly in European countries (8–12).

The development of this specific allergy to GSM does not always rely on earlier sensitization to CM proteins as only 19% of the GSM-allergic/CM-tolerant patients had suffered from an outgrown CM allergy (12). Moreover, CM-allergic children successfully treated with oral immunotherapy can remain allergic to GSM protein (13–15). Induction of GM-allergy through skin exposure has been also reported (16, 17). Allergy to GSM is frequently associated with severe symptoms even after ingestion of low amount of caprine or ovine milk protein in complex food matrices (11, 18–21). Beyond the mandatory labeling in EU for foods containing milk protein, the French Agency for Food, Environmental, and Occupational Health and Safety (ANSES) suggested to update the list of allergenic ingredients by adding emerging allergens such as milk from small ruminants (22). Allergen risk management must then assess the unintentional presence of GSM protein in food matrices, including contamination in production lines for dairy products. This assessment thus requires the development of sensitive methods able to detect specifically GSM protein contaminations along the entire food chain process.

Whole Casein (CN) constitutes up to 80% of the GSM proteins and is present in various dairy products as well as ingredients in processed food matrices. Among the four components of CN, i.e., αS1, αS2, β, and κ-caseins, the β-casein (β-CN) constitutes ~36% of the whole bovine CN and up to 50–60% of the whole CN from GM and SM, depending on genetic polymorphisms of the small ruminants (6, 7, 23). The caprine and ovine β-CN are 99% identical while the sequence identity between the caprine and bovine β-CN is 92%. Nevertheless, linear IgE-binding epitopes are not necessarily conserved between the caprine and bovine homologs. We previously showed that patients allergic to GSM and tolerant to CM display an IgE response to the caprine and ovine β-CN without significant cross-reactivity to the bovine homolog (8, 24). Specific IgE-binding to the caprine β-CN was mostly restricted to two domains, corresponding to amino acid residues 44–88 and 130–178 (25). Among the substitutions occurring between the caprine and bovine β-CN sequences, we further identified the critical role of the substitution T63P in the major non-cross reactive IgE-binding epitope of caprine β-CN (25). We also produced monoclonal antibodies (mAb), which were highly specific to the caprine β-CN.

In the present work, we aimed to develop an ELISA for the specific detection of the caprine β-CN, irrespective of the presence of CM proteins in the food matrix. The test was evaluated for its performances (sensitivity and specificity) with various processed dairy foods, in relation to their IgE reactivity. We finally assessed the assay sensitivity for the detection of GM contaminations in CM.

## Materials and Methods

### Anti-β-CN mAb Production and Characterization

Antibodies specific to caprine caseins were produced in mice tolerized to whole bovine CN through the oral route and then sensitized to whole caprine CN as previously described (25). In parallel, cross-reactive mAbs to β-CN were obtained from mice sensitized to bovine CN. All experiments were performed in compliance with the French and European regulations on care and protection of Laboratory Animals (EC Directive 86/609, French Law 2001-486, June 6, 2001) with permission 91–493 of French Veterinary Services. The laboratory animal facility care was approved by the French Veterinary Services and CEA agreement D-91-272-106 from the Veterinary Inspection Department of Essonne (France).

Spleen cells from mice producing the highest levels of IgG1 to CN were fused with NS1 mouse myeloma cells. The antibody-secreting cells (hybridomas) were screened by analyzing cell supernatants on microtiter plates coated with either bovine or caprine β-CN and using acetylcholinesterase (AChE)-labeled goat anti-mouse antibodies (Jackson ImmunoResearch, Europe Ltd.) as tracer (26).

Selected hybridoma cells were cloned and expanded as ascitic fluids in BALB/c mice. Specificity of the mAb purified from ascitic fluids by protein A affinity chromatography was fully characterized using recombinant, plasmin-derived, and synthetic peptides of bovine and caprine β-CN as previously described for human IgE-binding studies (24, 25). Briefly, microtiter plates were coated with either native, recombinant β-CN or peptide at 5 μg/mL in 50 mM carbonate buffer pH 9.2. Before use, plates were washed and each well was filled with 50 μL of purified mAb over a concentration range from 0.001 to 1 μg/mL in EIA buffer [0.1 M phosphate buffer, 0.1% bovine serum albumin free of protease and immunoglobulins (Sigma-Aldrich, St-Louis, USA), 0.15M NaCl, 0.01% sodium azide, pH 7.4]. After 2 h of incubation at room temperature (RT), plates were washed and each well was filled with 50 μl of goat anti-mouse AChE conjugate. After 2 h at RT, wells were washed, filled with 200 μL of Ellman's reagent and the absorbance was measured at 414 nm.

### Development of Anti-caprine β-CN Two-Site Immunometric Assays

Anti-caprine β-CN mAb was immobilized on microtiter plates (Immunoplate Maxisorp®, Nunc) at 5 μg/mL in 50 mM phosphate buffer, pH 7.4, for 24 h at 4°C. Plates were then washed and saturated for at least 4 h at RT using EIA buffer. Diluted samples or internal standards were added (50 μL/well) and incubated for 2 h at RT under agitation. Wells were washed and 50 μL of biotinylated anti- β-CN mAb (antibody:biotin molar ratio 1:20, EZ-link® NHS-PEG4-biotin, Thermo Scientific) were dispatched to each well at a concentration of 100 ng/mL in EIA buffer. After 2 h at RT, wells were washed and 50 μL of AChE-labeled streptavidin were dispensed for 20 min at RT. After washing, Ellman's reagent was added and absorbance was measured at 414 nm. Mean blank and SD blank were estimated by measuring eight replicates of signals obtained with EIA buffer. The limit of detection (LoD) and of quantification (LoQ) were estimated as mean_blank_ + 3 × SD_blank_ and mean_blank_ + 10 × SD_blank_, respectively. Precision was characterized by determining intra- and inter-assay coefficients of variation CV. Intra-assay variation was estimated by measuring different concentrations of native caprine β-CN standards eight times on a single plate (range from 0.156 to 200 ng/mL). The working range was then defined as the concentration range for which an intra-assay CV <10% was obtained. The inter-assay CV was calculated with two concentrations of standard inside the working range routinely assayed on different plates. Assay specificity was assessed by measuring signals in the concentration range from 0.1 to 10 μg/mL of purified proteins from CM, GM, and SM (8, 24). Cross-reactivity coefficients (in %) were calculated as the ratio of standard concentration divided by cross-reactant concentration providing the same signal in the two-site immunometric assays.

### Preparation of Samples From Processed Dairy Products

Dairy products classically found in France were purchased from retail outlets. They comprised semi-skimmed UHT milks (Lactel), yogurts (Danone, Vrai), soft goat cheese (Soignon) and semi-soft cheeses (Mini-Babybel cow cheese and Mini-Babybel goat cheese containing 10% of GM). Raw milks from one cow and one goat were included as control.

Yogurt or cheese (1 g) were resuspended in 10 mL of 50 mM ammonium carbonate buffer, 10 mM EDTA, 1% Octyl-beta-Glucoside using DT-20 dispersing tube (IKA® ULTRA-TURRAX®). After homogenization, samples were mixed during 1 h at 4°c, aliquoted and stored at −80°C. Samples were diluted in the ammonium carbonate buffer at 1 mg of protein/mL by taking into account the initial protein content of the dairy product.

### IgE-Binding Capacity as Determined by Reverse East Inhibition

IgE binding capacity was performed in a reverse EAST inhibition assay using sera from retrospective studies (24, 25). One serum from CM-allergic patient (#116) and two sera from GM-allergic/CM tolerant (#64, #183) patients were selected for this analysis. Anaphylactic shock was reported for the CM-allergic patient after ingestion of bovine milk product. His serum displayed an IgE response above 50 IU/mL for both bovine and caprine β-CNs. GM-allergic/CM tolerant (#64, #183) patients suffered from angio-oedema or asthma, urticaria and rhino-conjunctivitis, respectively, after ingesting goat cheese. Specific IgE levels were 10 and 50 IU/mL for caprine β-CN and negative or below 2 IU/mL for bovine β-CN, respectively.

For reverse EAST inhibition, plates were coated with anti-human IgE mAb LE27. Fifty μL/well of serum from each patient at appropriate dilutions (1:100 to 1:400) were incubated overnight at 4°C. After washing, 25 μL of inhibitors (i.e., increasing concentrations of proteins from dairy samples) and 25 μL of AChE-labeled caprine β-CN were mixed and incubated for 4 h at RT. Results were expressed as B/B0, where B0 and B represent the amount of labeled caprine β-CN bound to immobilized IgE antibodies in the absence or presence of a known concentration of inhibitor, respectively.

### Spiking Experiments

One mL of raw GM was diluted in 10 mL of raw CM corresponding to a contamination of 100,000 ppm. Ten-fold serial dilutions (1 in 10 mL) were then performed in CM to finally obtain a solution of 10 ppm of GM in CM. This last dilution was used to generate a CM contaminated at 2 and 1 ppm of GM. Spiking experiment was similarly performed with UHT CM spiked with UHT GM.

For two-site immunometric assays, the samples were two-fold diluted in EIA buffer with 0.1% Tween® 20 Detergent before analysis. Mean blank and SD blank were estimated by measuring replicates of signals obtained with EIA buffer, 0.1% Tween® 20. The signal-to-noise ratio was evaluated in comparison with CM milk.

## Results

### Specificity of Anti-β-CN mAb

Four hybridomas producing mAb specific to the caprine β-CN and not reacting to the bovine homolog were selected, cloned, expanded and purified. The mAb CC1 and CC7B recognized the C-terminal part (f108-207) of the caprine β-CN (Table 1). The sole substitution of the Lys residue at position 132 of the recombinant caprine β-CN by the Asn residue found in the bovine counterpart abolished their binding. The mAb SCB1 and SCB4 recognized the N-terminal fragment f(29-107) of β-CN. These mAbs were able to bind to a synthetic peptide covering the domain 49–79 but not to the caprine β-CN carrying the sole substitution of Thr residue at position 63 by the corresponding bovine Pro residue.

**Table 1:** Specificity of anti-β-CN monoclonal antibodies.

			**mAb anti-β-casein**			
			**CC1/CC7**	**SCB1/SCB4**	**CC11**	**VB1**
Bovine	Native	β-casein	–	–	+	+
		f29–105	–	–	–	+
		f106–209	–	–	+	–
Caprine	Native	β-casein	+	+	+	+
		29–107	–	+	–	+
		108–207	+	–	+	–
	Synthetic peptide	f49–107	–	+	–	+
		f49–79	–	+	–	–
		f80–107	–	–	–	+
	Recombinant	rβcap	+	+	+	+
		rβcap T63P	+	–	+	+
		rβcap K132N	–	+	+	+

Two cross-reactive mAb, CC11 and VB1, were produced from mice sensitized to the bovine CN. The mAb CC1 bound to the fragments (f106–209) and (f108–207) covering the C-terminal part of the bovine and caprine β-CN, respectively. The mAb VB1 recognized the fragment (f29–105) of the bovine β-CN and the peptides (f29–107) and (f80–107) from the caprine β-CN.

### Development of Two-Site Immunometric Assays

A matched antibody pairs test was performed using each of the non-cross reactive mAb SCB1/SCB4 or CC1/CC7 as immobilized mAb to capture caprine β-CN. For the detection, in addition to a mAb specific to the caprine β-CN, we also tested cross-reactive mAb, i.e., mAb CC11 and VB1 that recognized both caprine and bovine β-CN.

**Figure 1:**
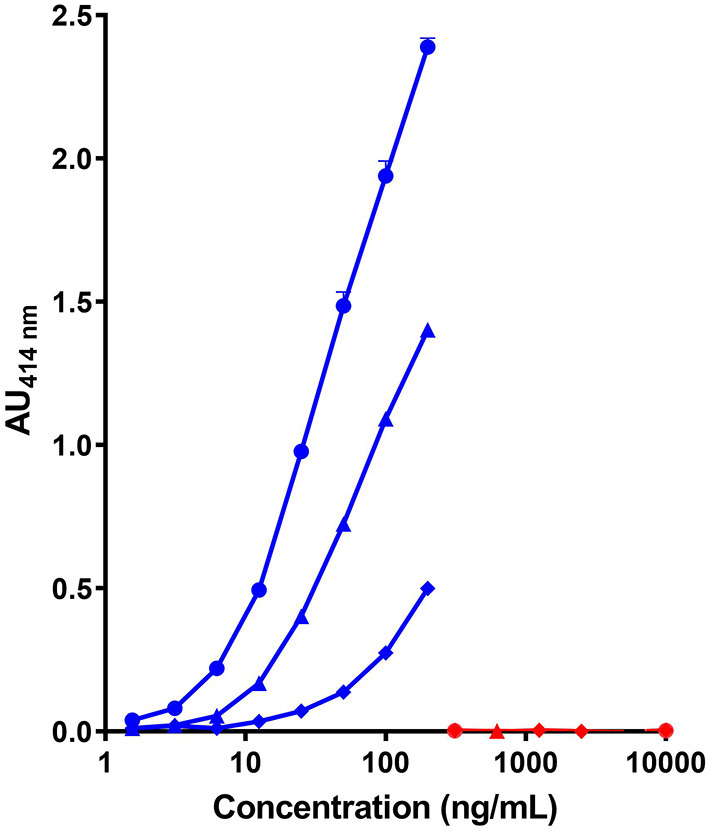
Detection of caprine (blue symbols) and bovine β-CN (red symbols) using three pairs of immobilized/biotinylated mAbs, (•)SCB1/CC11, (▴) SCB1/VB1, and (♦) CC7/SCB1 in corresponding two-site immunoassays.

As shown in Figure 1, the combination of the non-cross reactive mAb CC7 (or CC1) for the capture and mAb SCB1 (or SCB4) for the detection did not provide a highly sensitive assay of the caprine β-CN with a LoD above 10 ng/mL. Same characteristics using SCB1 for the capture and CC7 for the detection were obtained (data not shown). A higher level of sensitivity (LoD <5 ng/mL) was obtained by using mAb SCB1 for the capture and the cross-reactive mAb VB1 or CC11 for the detection. No significant signal with the bovine β-CN was observed with any of the tested pairs of mAb, even at a protein concentration up to 10 μg/mL.

### Characteristics of the Anti-caprine β-CN ELISA

Development of two-site immunometric assays was then pursued only with SCB1 as the capture mAb and VB1 or CC11 as the detection mAb. The two assays displayed very similar sensitivity and specificity (Table 2). A LoD between 1.6 and 3.2 ng/mL was achieved with purified caprine β-CN as standard reference. The other caprine caseins showed a cross-reactivity coefficient below 0.04% at a concentration up to 10 μg/mL. The signal above LoD obtained with the caprine β-lactoglobulin (BLG) was probably due to β-CN contamination in the purified BLG rather than to an actual cross-reactivity between caprine BLG and β-CN. Moreover, none of the tested CM proteins, including bovine β-CN and BLG, gave a significant signal at a concentration up to 10 μg/mL. In contrast, a cross-reactivity close to 100% was observed between caprine and ovine β-CN in the two immunometric assays.

**Table 2:** Characteristics of the two-site immunometric assays for caprine β-CN detection.

	**Capture mAb/biotinylated mAb**	
	**SCB1/VB1**	**SCB1/CC11**
**Caprine** **β-casein**		
LoD (Mean of all blank values + 3 × Standard deviation) (ng/mL)	3.2	1.6
LoQ (Mean of all blank values + 10 × Standard deviation) (ng/mL)	4	3.2
Working range (ng/mL)^*^	6-200	4-200
Cross-reactivity (%)^**^		
Caprine^***^		
αs1-casein	<0.032	<0.016
αs2-casein	<0.032	<0.016
κ-casein	<0.032	<0.016
β-Lactoglobulin	0.1	0.15
Bovine^***^		
β-casein	<0.032	<0.016
αs1-casein	<0.032	<0.016
αs2-casein	<0.032	<0.016
κ-casein	<0.032	<0.016
β-Lactoglobulin	<0.032	<0.016
Ovine^***^		
β-casein	98	102

* *The working range was defined as the range with an intra-assay CV < 10%*.

** *The cross-reactivity coefficients were determined as the ratio of caprine β-casein concentration divided by cross-reactant concentration providing the same signal in the two-site immunometric assay. <0.032 or <0.016: no significant signal was detected at the highest concentration of 10 μg/mL*.

*** *Milk proteins were purified as previously described (8)*.

### IgE-Binding Capacity of Processed Dairy Products

**Figure 2:**
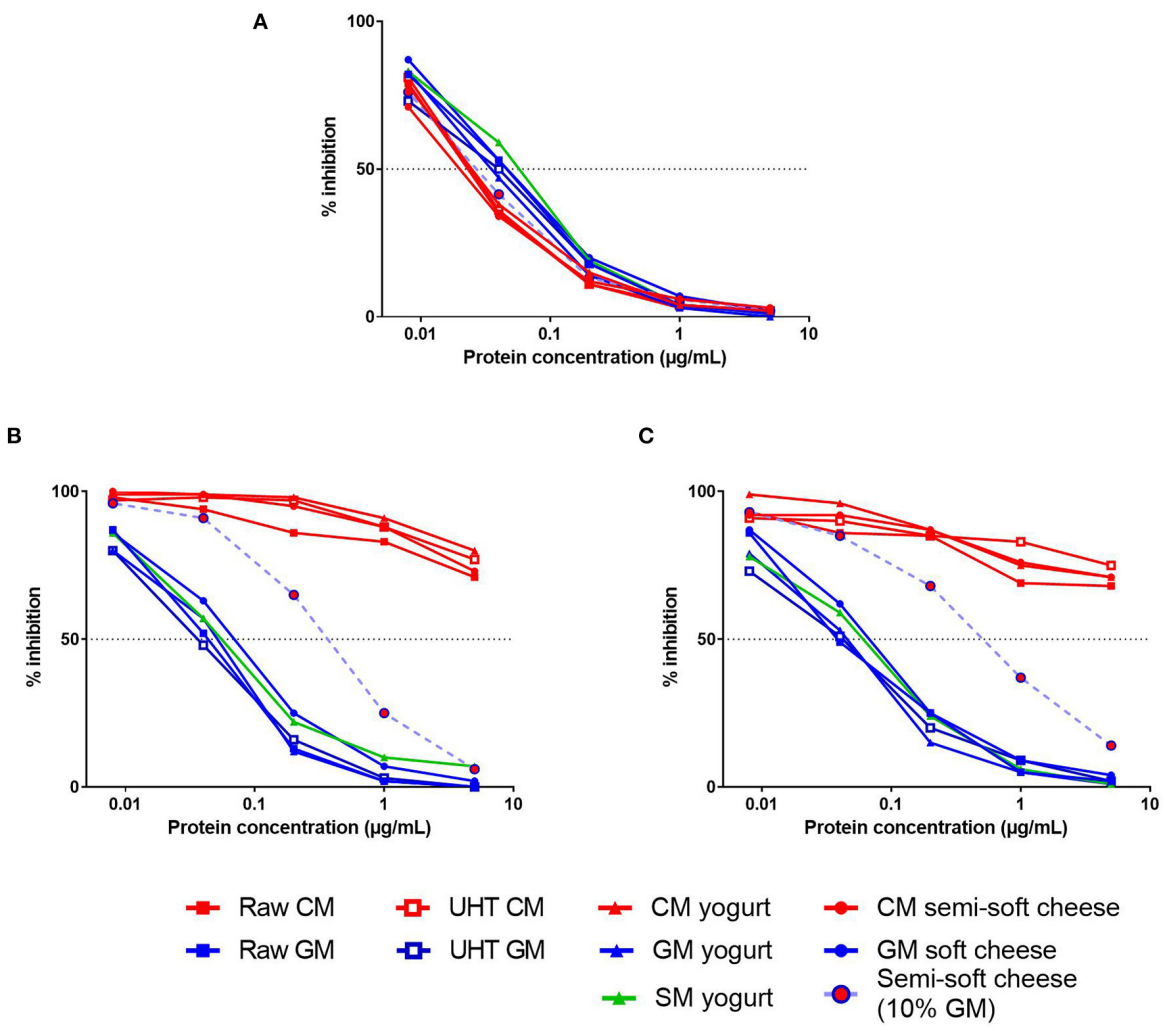
Competitive inhibition of IgE-binding (reverse EAST inhibition) to caprine β-CN by increasing concentrations of various dairy products (see legends) by using sera from CM-allergic patient **(A)** and from GM-allergic/CM-tolerant patients **(B,C)**.

We first evaluated the IgE-reactivity of β-CN present in protein extracts from different dairy products, including raw and heat-treated milk, yogurts, and cheeses (Figure 2). Using a serum from a CM-allergic patient, all CM products exhibited comparable capacities to inhibit the IgE-binding to the caprine β-CN, thus illustrating the expected IgE cross-reactivity between bovine and caprine CN (Figure 2A). These results also indicated that the different processes, i.e., heat treatment, fermentation and cheese production, did not alter significantly the bovine β-CN immunoreactivity. Similarly, no significant difference was observed between β-CN immunoreactivities from the different products containing GSM protein although GSM products displayed a slightly lower inhibitory capacity than the CM products.

Conversely, a very low IgE cross-reactivity between CM and GSM products was observed when using sera from two GM-allergic/CM-tolerant patients (Figures 2B,C). The processed GSM products displayed a capacity to inhibit IgE-binding to the caprine β-CN very similar to that of raw GM. In contrast, CM products failed to inhibit more than 30% of the IgE-binding at the highest tested protein concentration. The cheese containing 90% of CM and 10% of GM exhibited a half-maximal inhibitory concentration about 10-fold higher than that of the cheese containing only GM.

### Detection of Caprine β-CN in Processed Dairy Foods

**Figure 3:**
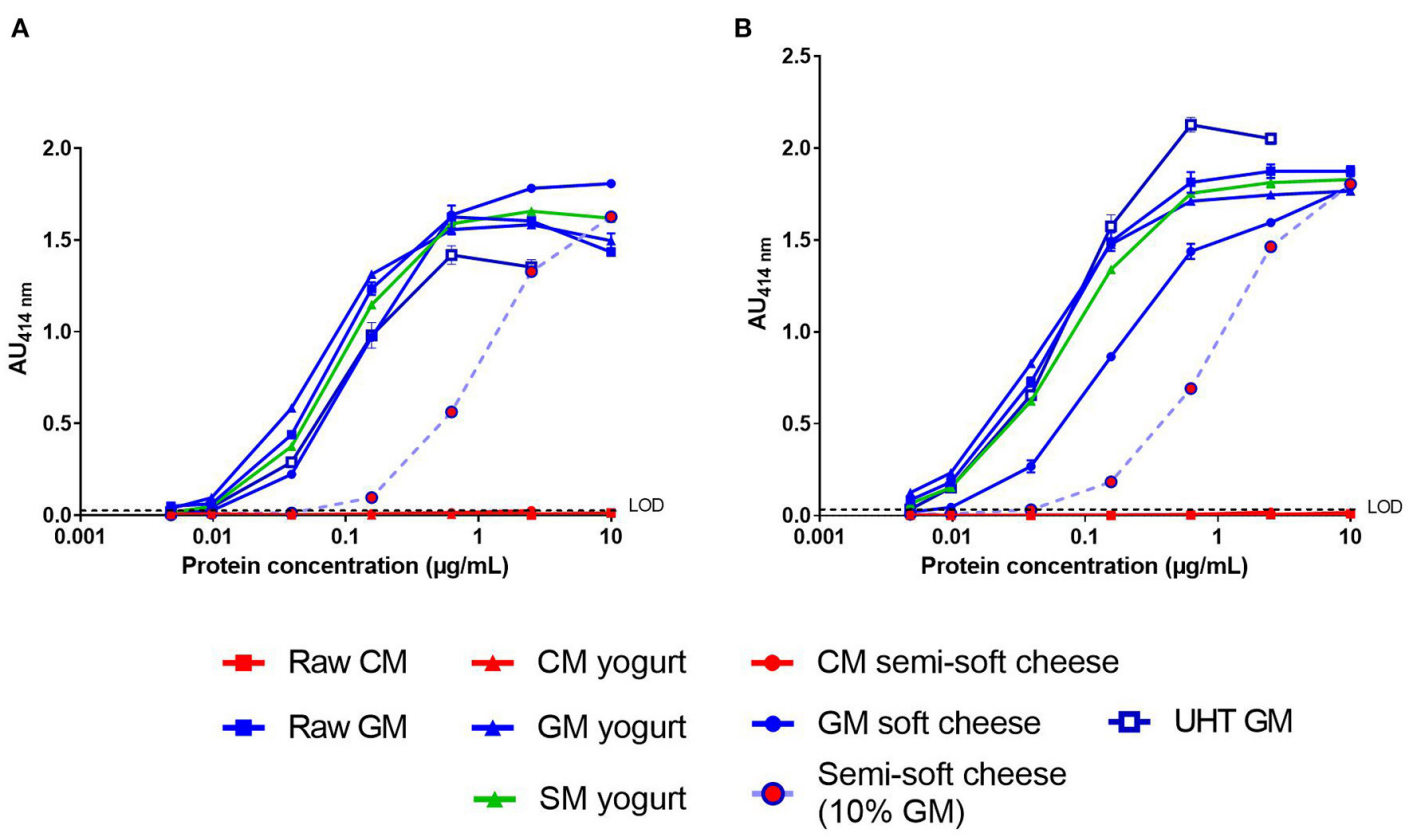
Detection of caprine β-CN in various dairy products (see legends) using SCB1/VB1 **(A)** or SCB1/CC11 **(B)** two-site immunoassay. LoD, Limit of Detection.

We then analyzed the processed dairy products using the two-site immunoassays with the matched mAb pair SCB1/VB1 or SCB1/CC11 (Figure 3). No significant signal was observed in the CM products, regardless of the mAb pair tested (Figures 3A,B). For both assays, the dose-response curves obtained with GSM products were parallel to that observed with raw GM. The LoD of caprine β-CN in GSM products was around 10 ng/mL for the SCB1/VB1 pair (Figure 3A). A lower LoD of 5 ng/mL was reached with the mAb SCB1/CC11 pair except for the GM soft cheese (Figure 3B). Both assays detected a level of caprine β-CN in the GM semi-soft cheese corresponding to a GM concentration of around 10%. Furthermore, dose-response curves from goat's and sheep's yogurts overlapped.

### Detection of GM Contamination in CM

**Figure 4:**
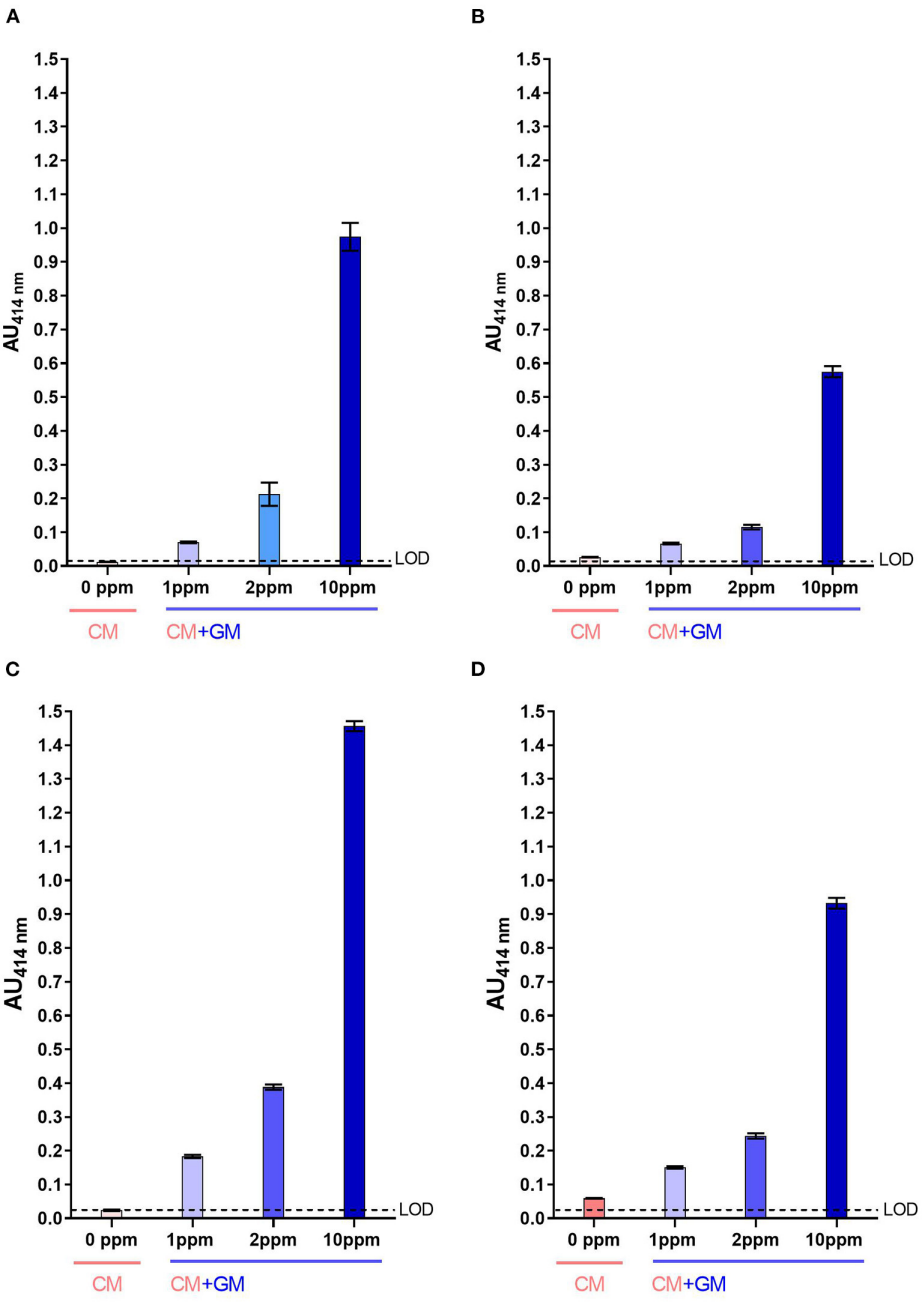
Detection of caprine β-CN in CM spiked with GM using SCB1/VB1 **(A,B)** and SCB1/CC11 **(C,D)** two-site immunoassays. Analysis was performed using raw milk **(A,C)** and UHT milk **(B,D)**. LoD, Limit of Detection.

For both two-site immunoassays, signals measured with raw CM were below the LoD (Figures 4A,C) while those measured with heat-treated CM were slightly above the LoD, in particular with the mAb pair SCB1/CC11 (Figures 4B,D). Whatever, caprine β-CN was significantly detected in raw CM spiked with raw GM at a contamination level as low as 1 ppm, with a signal-to-noise ratio of at least 3:1. For UHT milk, signals measured for a GM contamination of 1 ppm were almost two-fold higher than those measured with pure CM.

## Discussion

Detection of allergen contaminations in food matrices is one of the keystones of allergen risk management. Unintentional cross-contamination with milks from different ruminants may in particular arise during the processing of dairy products. Various methods including biochemical, immunochemical and molecular biology techniques have been developed to discriminate between milk proteins from different ruminants (27–30). These methods are applied mostly for the detection of milk adulteration, i.e., the fraudulent incorporation of CM in GSM for economic reasons. Although some of these methods could also detect GSM presence in CM, they do not offer a sensitivity sufficient to detect trace contaminations. In this regard, two-site immunoassay remains the method of choice for its specificity, sensitivity and feasibility. However, production of antibodies specific to a milk protein from a ruminant without any cross-reactivity to homologs from other ruminants is particularly challenging because of the high sequence identity among ruminants' milk proteins. One approach to obtain specific antibodies relies on the immunization of animals with peptides covering domains displaying the highest structural differences between the milk proteins of interest (30). However, antibodies obtained through this approach often exhibit a lower affinity to the native full protein than to the targeted peptide, thus limiting the sensitivity and rendering the assay inadequate for the detection of trace amount of protein contamination (31, 32). We thus applied another strategy by inducing tolerance to bovine CN in mice before sensitizing them to the caprine CN in order to limit the production of cross-reactive antibodies. This strategy was inspired by a study reporting that CM-allergic patients could remain allergic to GM after a successful desensitization to CM (14). Using this approach, we succeeded to obtain different mAbs specific to caprine αS1-, β-, or κ-CN.

We then chose to target specifically the β-CN for its high IgE immunoreactivity and because it is generally the most abundant CN in GSM (23, 33). Moreover, this casein is only slightly hydrolyzed in cheese by chymosin and/or pepsin (34–36). In addition, β-CN is generally considered to possess no or little secondary structures and then to be heat-stable (37–39). This property was confirmed in the present study since the IgE-binding capacity of β-CN was not affected in the different processed milk products. Indeed, the IgE-binding epitopes of caprine β-CN were maintained during pasteurization and UHT processing as previously observed for CM and GM caseins (40–43). Moreover, the IgE-binding capacity of caprine β-CN was maintained in dairy products even after enzymatic processes that occur during ripening, in accordance with the allergenicity of CM and/or GM cheeses (9, 10, 44).

The mAb recognizing the caprine β-CN, with or without cross-reactivity with the bovine β-CN, were directed against four different domains. Although the pair of non-cross-reactive mAb CC7 and SCB1 was functional to detect caprine β-CN, a higher sensitivity without decrease of specificity was obtained by using for detection a cross-reactive mAb, i.e., mAb VB1 or CC11. Interestingly, the mAbs SCB1/VB1 pair recognized the short domain 49-107 of β-CN, which encompasses major IgE-binding epitopes (24). As observed for the IgE-reactivity, heat-treatment, yogurt fermentation, casein coagulation, and cheese ripening generally did not affect the detection of caprine β-CN by our two-site immunometric assays. Thus, although protein aggregation during heat treatment could reduce the epitope accessibility and thereby the β-CN detection, the assay was still able to detect a contamination of 2 μL of UHT GM diluted in 1 L of UHT CM. Of note, detection of β-CN was partially altered in soft cheese for the mAbs SCB1/CC11 pair. Soft cheese processing could probably generate limited proteolysis and breakdown products of caprine β-CN not recognized by the SCB1/CC11 pair but still detectable by the SCB1/VB1 pair. This result suggests that the domain 49–107, recognized by the pair SCB1/VB1, is more resistant to chymosin and pepsin proteolysis during renneting than the larger fragment recognized by the pair SCB1/CC11. This assay could then allow the detection of caprine β-CN and derived peptides in multiple dairy products in spite of proteolytic processes including possible hydrolysis by plasmin (35, 45).

Considering a LoD of 10 ng/mL of caprine β-CN in dairy products resuspended with a mass-to-volume ratio of 1:10, a LoD of 0.1 ppm (100 ng of β-CN in 1 g of dairy matrix) was reached without significant interference from bovine milk. The LoD of our immunoassays was then at least as sensitive as the ones developed to assess CM protein contamination, in particular to detect bovine casein (46, 47). Unfortunately, eliciting doses of GSM based on clinical data are still missing, probably because of the low prevalence of GSM allergy without CM allergy. Allergic reactions have been described after ingestion of limited amounts (few grams) but without clear quantitative eliciting doses (8). Two cases of allergic reaction were observed due to the ingestion of foods contaminated with GM protein along the food chain (8). Moreover, 15 allergic patients treated with cow's milk oral immunotherapy reacted to cumulative doses varying between 5 and 100 g of GM or SM cheese during open food challenge (14). Clinical studies are still then required to establish threshold doses for GSM allergy. Nevertheless, assuming similar threshold doses for CM and GSM allergies, the present tests could provide a high enough sensitivity to warrant the safety of food products for allergic patients.

## Conclusion

In the present work, we developed two immunoassays allowing the detection of caprine β-CN with a LoD lower than 4 ng/mL and without any cross-reactivity with the bovine β-CN. These in-house immunoassays were both able to detect GM contamination in CM at a level as low as 1–2 ppm. They are also effective for the detection of caprine β-CN in dairy products whatever the processes used, i.e., pasteurization, UHT treatment, fermentation, casein coagulation, and cheese ripening, in line with IgE-immunoreactivity of the tested products.

## Data Availability Statement

The raw data supporting the conclusions of this article will be made available by the authors, without undue reservation.

## Ethics Statement

The animal study was reviewed and approved by the French Veterinary Services and CEA agreement D-91-272-106 from the Veterinary Inspection Department of Essonne (France). All experiments were performed in compliance with the French and European regulations on care and protection of Laboratory Animals (EC Directive 86/609, French Law 2001-486, June 6, 2001) with permission 91–493 of French Veterinary Services.

## Author Contributions

HB and SH designed the whole study, performed the experiments, interpreted the data, and wrote the manuscript. NG help to perform some experiments. KA-P managed the study and critically revised the manuscript. All authors approved the submitted version.

## Funding

This study obtained a financial support from INRAE (Institut National de Recherche pour l'Agriculture, l'Alimentation et l'Environnement).

## Conflict of Interest

The authors declare that the research was conducted in the absence of any commercial or financial relationships that could be construed as a potential conflict of interest.

## Publisher's Note

All claims expressed in this article are solely those of the authors and do not necessarily represent those of their affiliated organizations, or those of the publisher, the editors and the reviewers. Any product that may be evaluated in this article, or claim that may be made by its manufacturer, is not guaranteed or endorsed by the publisher.

## Abbreviations

CN: casein
CM: Cow's Milk
GM: Goat's Milk
SM: Sheep's Milk
GSM: Goat's and Sheep's milks
mAb: monoclonal Antibody
RT: Room Temperature.
